# Congenital Malformation—Extract of Belladonna in Mammary Inflammation

**Published:** 1862-01

**Authors:** J. J. Lescher

**Affiliations:** Mount Carmel, Ills.


### Correspondence.

### Congenital Malformation—Extract of Belladonna in Mammary Inflammation.

TO THE EDITORS OF THE CHICAGO MEDICAL EXAMINER.

Gentlemen:—On the 24th of September, 1859, I delivered Mrs. Keppell, a German, of a large and healthy female child, at full term. The day following, the mother in great trepidation, remarked to me, "the baby isn't right, it stools the wrong way! O what will become of it!" Upon an inspection, an anomalous condition of the genitals presented itself, which at the time, struck me as unique, the like of which had never passed under my notice, neither can I find a parallel case on record, so far as my observation extends.

The perineum presented nothing unusual, except that, in place of the anal opening at the posterior termination of the raphe, there was a teat-like prominence occupying the point where the rectum should have terminated. Without disturbing the parts, the entrance to the digestive tube was invisible; but upon separating the labia pudendi, I discovered, anterior to the posterior commissure, occupying the space of the fossa naviculare, a small opening communicating with the rectum, which at first view, I regarded as fistulous, supposing the case to be one of imperforate anus. A close investigation, however, revealed the fact, that the opening was provided with a perfect sphincter, both in appearance and in function—that it was, in truth, the anus!

The external sphincter is perfect in all its characteristics, except that it seems less developed, in size, or more unyielding, inasmuch as its capacity for expansion or distension is considerably less than it should be, presenting in consequence, a greater obstacle or resistance to the passage of the contents of the bowel. So far as can be discovered, with the exception of this malformation, the child is perfect in all its parts, and is now large and thrifty, showing that the little difficulty attending defecation, does not interfere in any degree, with its welfare and growth.

When about four months old, defecation suddenly became so difficult, that but very small quantities could be discharged, causing the infant great uneasiness; finally the feces were entirely arrested for several days, causing some fever and much suffering, when I was sent for. I found the abdomen large and distended, seemingly to its utmost capacity. Separating the labia majora, and bringing into view the pudendal anus, I discovered a hard smooth substance infringing upon the anus from within, which I suspected to be a pebble; mentioning my suspicions, the mother observed that the child had been playing with pebbles sometime prior to the date of its fretfulness; one of which it must have swallowed, and proving too large for expulsion, it had lodged within the external sphincter, effectually blocking up the passage. I bore against it with a probe, pushing it upward, whereupon the feces passed freely, so long as I held the pebble upon the point of the probe. After a large evacuation, supposing the bowels pretty well emptied, I withdrew the probe, and attempted the introduction of the spoon-shaped end of a grooved director, with a view to engage the pebble in the scoop, that I might remove it. The constriction of the sphincter, however, was so considerable, that its introduction was attended with difficulty, causing the parts to bleed. Failing with the director, I bent a probe, introducing the hooked end into the rectum, but could not engage the pebble, as it would slip upward, in spite of my efforts. In each attempt to get rid of the foreign substance, large quantities of feces would escape. The pebble acted as a perfect valve. I suggested to the mother, that it could only be removed, at that time, by incising the sphincter, to which she objected so strongly, that I did not feel at liberty to press the matter, believing that its presence could do no other harm than to mechanically obstruct the passage, which might be obviated by the repeated use of the probe.

The infant, as may be imagined, was "itself again," after its bowel was relieved of its contents. For a considerable length of time, the mother made frequent use of a probe, for the purpose above mentioned, enabling the child to free its bowel at regular intervals, preventing any fecal accumulation.

Until quite recently, I saw no more of the child, when, upon inquiry, I ascertained that the pebble, that had played such singular freaks, had in all probability been dislodged, as it had not discovered itself for some months. The only difficulty now, seems to be, that unless the stooling is semi-fluid, it is voided with considerable straining.

Growing out of this singular malformation, an interesting question presents itself to my mind. It must be remembered, that the child is purely Teutonic, both father and mother Germans, with large heads and broad shoulders. Should the child mature to womanhood and maternity, more especially if wedded to a German, could she give birth to a child without a laceration of the recto-vaginal wall, involving the anus? Would there not be infinitely greater danger of this accident, than if this malformation did not exist?

Would a laceration—particularly in the first labor, and if in the first, in every subsequent one,—be avoidable?

If the fœtus were above the medium size, as might be presumed, for the reasons already stated, would not laceration be inevitable?

---

Although not pertinent to the foregoing subject, I cannot refrain in this connection, from adding my testimony to the recorded efficacy of the local application of a watery solution of the Ext. Belladonna in mammary inflammation, to which my attention was first directed in 1858, through an English translation, by Prof. W. H. Byford, of Chicago, of an article from a German periodical, which appeared in the *North-Western Medical and Surgical Journal.*

In her first and second confinements, Mrs. K. had suffered excruciatingly from abscess of one breast. I was in attendance when confined with her third child. Knowing her liability to inflammation and its fearful consequences, of her "sore breast," as she called the mamma which had caused her such exceeding agony during each prior confinement, and which had become indurated in consequence, and partly unfitted for lactation, I was very desirous of averting an evil which seemed imminent.

Upon this occasion, she was delivered without any untoward symptoms, early on the morning of September 15th, 1858; on the third day following, she was seized with a severe and prolonged chill, followed by fever. Both breasts were painful and tumid, but the "sore breast" was exceedingly tender and rapidly inflamed. Apprehensive that suppuration was inevitable, I fortunately bethought myself of the translation above referred to; in addition to a cathartic and antimony, I directed the breast to be bathed with a watery solution of the extract, in the proportion of ℈ss to the ounce, and the breast afterwards covered with a flannel cloth, moistened with the solution, with orders that the cloth be kept constantly moistened with it. To the great relief of the frightened woman, and my utter astonishment, every vestige of the inflammation disappeared in a few days,—without suppuration, of course.

I am not positive whether she gave suck with her "sore breast," or whether she "dried it up," though I am inclined to the opinion that it secreted a small proportion of milk.

In her last confinement, she escaped without any other trouble than a mild milk-fever, her "sore breast" more sensitive than the other.

Since my first trial of the extract of belladonna, I have had repeated opportunities of confirming my good opinion of this agent. With but one or two exceptions, entire success followed its application.

I might give the details of several cases, wherein its timely use saved them lancings and counter-openings, whilst in prior confinements, they had endured all the tortures of abscess. One case particularly arrests my attention:—

Some fifteen years ago, my sister, then residing in a neighboring village, some twenty miles hence, was confined with her first child. From some cause or other, inflammation and abscess of both breasts ensued, and, as is too often the case, poulticing was relied on to "bring it to a head." As might be expected, the pus burrowed between the lobes and underneath the integument, seeking exit through a number of openings. Months elapsed before she recovered, with the loss of her child. Using the ordinary means to prevent a similar occurrence during her second, third and fourth confinements, suppuration, more or less extensive, ensued each time. Before the fifth confinement, I had a solution of the extract in readiness, with instructions to apply it freely immediately upon the access of inflammation. The result proved entirely satisfactory; no abscess followed.

Last summer, she was again confined, and threatened with inflamed breasts, but a timely use of the remedy, arrested it speedily. J. J. LESCHER, M. D.

MOUNT CARMEL, ILLS., Dec., 1861.

